# RETRACTION: The Effect of Dexmedetomidine on Postoperative Wound Healing in Neurosurgical Patients: A Meta‐Analysis

**DOI:** 10.1111/iwj.70576

**Published:** 2025-04-21

## Abstract

**RETRACTION:** ZhuC. , CaoD. , ChenL. , ZhengX. , and LinD. , “The Effect of Dexmedetomidine on Postoperative Wound Healing in Neurosurgical Patients: A Meta‐Analysis,” International Wound Journal 21, no. 4 (2024): e14585, 10.1111/iwj.14585.38148721 PMC10961872

The above article, published online on 26 December 2023, in Wiley Online Library (http://onlinelibrary.wiley.com/), has been retracted by agreement between the journal Editor in Chief, Professor Keith Harding; and John Wiley & Sons Ltd. Following an investigation by the publisher, all parties have concluded that this article was accepted solely on the basis of a compromised peer review process. The editors have therefore decided to retract the article. The authors did not respond to our notice regarding the retraction.

**RETRACTION:** C. Zhu , D. Cao , L. Chen , X. Zheng , and D. Lin , “The Effect of Dexmedetomidine on Postoperative Wound Healing in Neurosurgical Patients: A Meta‐Analysis,” International Wound Journal 21, no. 4 (2024): e14585, 10.1111/iwj.14585.38148721 PMC10961872

The above article, published online on 26 December 2023, in Wiley Online Library (http://onlinelibrary.wiley.com/), has been retracted by agreement between the journal Editor in Chief, Professor Keith Harding; and John Wiley & Sons Ltd. Following an investigation by the publisher, all parties have concluded that this article was accepted solely on the basis of a compromised peer review process. The editors have therefore decided to retract the article. The authors did not respond to our notice regarding the retraction.

